# Prevention of violence against children in the home: an overview of reviews protocol

**DOI:** 10.1186/s13643-022-02153-6

**Published:** 2022-12-15

**Authors:** Jorge Cuartas, Dana McCoy, Hirokazu Yoshikawa, Michelle Bass, Ana Salazar, Nicholas Metheny, Felicia Knaul

**Affiliations:** 1 Harvard Graduate School of Education, 14 Appian Way, Cambridge, MA USA; 2 Centro de Estudios sobre Seguridad y Drogas (CESED), Universidad de los Andes, Cra 1 Nº 18A- 12, Bogotá, Colombia; 3 New York University Steinhardt & Global Ties for Children, New York, NY USA; 4 Countway Library, Harvard Medical School, Boston, USA; 5 Facultad de Educación, Universidad de los Andes, Bogotá, Colombia; 6 University of Miami, Coral Gables, USA

**Keywords:** Violence against children, Child maltreatment, Prevention, Interventions, Systematic review

## Abstract

**Background:**

Violence against children (VAC) in the home, or by household members, is a widespread problem with devastating and costly consequences for individuals and societies. In the past two decades, much research has been dedicated to the prevention of VAC in the home, in particular, in low- and- middle-income countries, but there are few systematic examinations of the growing body of literature. We present a protocol for an overview of reviews to synthesize the evidence from recent reviews on the effectiveness and characteristics of both universal and selective interventions to prevent VAC in the home or by household members.

**Methods:**

We will conduct an overview of reviews of quantitative studies of universal and selective interventions to prevent VAC in the home published after 2000. Our outcomes will be VAC in the home (e.g., physical, sexual, or psychological violence or neglect) and drivers or direct risk factors for VAC (e.g., beliefs or attitudes towards VAC, parenting stress). We will search for reviews in multiple databases using controlled vocabularies and keywords. We will use the AMSTAR 2 to assess the quality of reviews and will extract information on outcomes, main results, and geographic distribution of primary studies, among other data. We will conduct a narrative synthesis of the findings.

**Discussion:**

The proposed overview will provide timely evidence on effective strategies to prevent VAC in the home and will identify the key strengths and limitations of the current body of evidence on this topic. In doing so, we will inform future research, policy, and practice aimed at building effective strategies to prevent VAC globally.

**Systematic review registration:**

PROSPERO CRD42022304784.

**Supplementary Information:**

The online version contains supplementary material available at 10.1186/s13643-022-02153-6.

## Background

Violence against children (VAC) is a widespread problem with devastating and costly consequences for individuals and societies [1]. The World Health Organization defines VAC as the intentional use of physical force or power, threatened or actual, against people under 18 years old that results in, or has a likelihood of resulting in, harm, death, psychological injury, maldevelopment, or deprivation [2]. Some forms of VAC are physical maltreatment (including physical punishment, like spanking), emotional or psychological violence, sexual abuse, witnessing intimate partner violence, and neglect or abandonment (see Table 1 for further examples) [2]. Before COVID-19, up to 1 billion children aged 2–17 years experienced physical, sexual, or emotional violence or neglect in the preceding 12 months [3], and about 220.4 million—or 2 out of 3—children aged 2 to 4 years around the globe were exposed to physical punishment in the home [4]. Growing evidence indicates that VAC has increased amid the COVID-19 pandemic [5]. While VAC occurs in several settings, the home is the place where children experience physical, psychological, sexual, and other forms of violence most often [6, 7].

**Table 1:** Some forms of VAC in the home or perpetrated by household members

Form of VAC	Examples
Physical violence	Spanking, hitting with objects, other forms of physical punishment, shaking, burning, kicking, biting, forcing the child to stay in uncomfortable positions
Sexual violence	Rape, unwanted sexual advances, sexual harassment, forced marriage
Psychological or emotional violence	Hostility, threatening, yelling, calling the child by offensive names, constant rejection
Witnessing violence	Witnessing intimate partner violence
Neglect	Social deprivation, abandonment, psychosocial deprivation

Decades of research in the fields of public health, neuroscience, psychology, sociology, and economics have demonstrated that any form of VAC has the potential to interfere with children’s health, development, and well-being. For individuals, VAC can alter the development of brain architecture [8, 9], impair cognitive and social-emotional development [10, 11], and increase the risk for both physical and mental health disorders [12, 13]. At a societal level, the estimated cost of VAC (exclusively considering the victims) has been estimated to correspond to approximately 8 to 12% of the global Gross Domestic Product [14–16]. Furthermore, VAC is a violation of children’s rights [17] and a major barrier to the global policy objectives established in the Sustainable Development Goals (SDGs) [18].

Considering the high prevalence and economic costs of VAC, there is an urgent need for effective strategies to prevent VAC. These should begin in the home, where VAC is most prevalent. In response, the United Nations (UN) has proposed several strategies for ending VAC, including the INSPIRE framework [2]. The INSPIRE framework uses a social-ecological perspective, acknowledging that the multiple contextual drivers of VAC in the home require systemic and multilevel interventions. The framework proposes the implementation and enforcement of laws, change in harmful norms and values, promotion of safe environments, parent and caregiver support, income and economic strengthening, response and support services, and education and life skills as promising strategies to end VAC in the home [2]. Despite these policy efforts, the Global Status Report on Preventing Violence Against Children 2020 [19] concludes that while many countries have implemented strategies to prevent VAC in the home, few have plans that include measurable targets, making it difficult to identify effective strategies that can be scaled up and out.

One overview of reviews previously synthesized evidence on universal (i.e., those that target the general population) and selective (i.e., those focused on people at higher risk interventions) that target VAC [20], offering evidence for some promising strategies to prevent VAC. In particular, the review concluded that home-visiting interventions, parent education, and multi-component interventions show promise in preventing VAC. The review also found that about 83% of studies were from the USA, and only 0.6% were from low- and middle-income countries, where more than 90% of children live [21]. Furthermore, the studies included in the review had significant methodological shortcomings, limiting our understanding of the effectiveness of interventions to prevent VAC. Finally, the review only considered studies published before 2009. Since 2009, other reviews of reviews have examined the prevention strategies for violence against girls [22], but we are not aware of other systematic reviews of reviews that focus on VAC in general. As such, there is a need for an updated systematic overview of reviews that collates evidence from the past two decades to inform future violence prevention efforts and identify gaps in the literature that can inform future research.

The current overview of reviews has three objectives: first, to synthesize evidence from reviews on the effectiveness and characteristics of both universal and selective interventions to prevent VAC in the home or by household members; second, to evaluate the methodological quality of the reviews; and third, to assess the geographic distribution and basic characteristics of the primary studies included in the reviews. Doing so, this study will identify the key gaps (e.g., methodological, geographic) in existing studies to inform future research on effective interventions to prevent VAC in the home. Furthermore, the review will provide evidence to inform policymakers and non-government organizations on the strategies to prevent VAC to protect children from all forms of violence and contribute to the achievement of the SDGs by 2030.

## Methods

### Protocol registration and reporting

This systematic review will include reviews of quantitative studies of universal and selective interventions to prevent VAC in the home or by household members. A systematic overview of reviews is defined as a systematic review that includes only reviews (e.g., rapid reviews, systematic reviews, meta-analysis) instead of primary studies, which seeks to summarize the published evidence on a broad research question [23]. We followed the Preferred Reporting Items for Systematic Reviews and Meta-Analysis for Protocols (PRISMA-P) to develop the protocol for this overview of reviews (see Additional file 1). This protocol was submitted to be registered in the International Prospective Register of Systematic Reviews (PROSPERO) on May 20, 2022.

### Inclusion and exclusion criteria

Table 2 summarizes the inclusion and exclusion criteria. The review will focus on an overview of reviews, systematic reviews, meta-analyses, and other reviews (e.g., rapid, scoping) of universal (i.e., aimed at the general population) and selective interventions (i.e., aimed at populations at higher risk) targeting parents and other caregivers to prevent (among other potential outcomes) VAC in the home or by household members published in peer-reviewed journals and gray literature. The review will include reviews that discuss randomized (i.e., experimental) and non-randomized quantitative studies (i.e., quasi-experimental, observational). The studies in the reviews could comprise pre-post comparisons or a comparison group (e.g., treatment and control group). The interventions that are the focus of the review must aim to prevent any or all of the following types of direct VAC in the home or by household members: physical (including physical punishment), sexual, psychological or emotional, and neglect perpetrated by someone over the age of 18 toward a child. The comparison could be to not receiving the intervention, a waitlist control, standard-of-care or other intervention, or a single sample measured pre- and post-intervention. This overview of reviews will consider two primary outcomes: (1) VAC in the home, as reported by parents, children, others, administrative records, and/or direct assessment, and (2) drivers or direct risk factors for VAC, including parents’ or caregivers’ knowledge, beliefs, and/or attitudes towards VAC (e.g., endorsement of physical punishment), parenting stress, low parenting confidence, and low self-regulation skills, among others. The overview of reviews will have no language or geographical restrictions. To provide new knowledge on the state of the science regarding the prevention of VAC, we will only include reviews published after 2000.

**Table 2:** Inclusion and exclusion criteria

	**Included**	**Excluded**
**Type of review**	• Systematic reviews • Meta-analyses • Other types of literature reviews	• Other types of publications (e.g., primary studies)
**Period**	• Published between 2000 and 2022	• Published before 2000
**Publication**	• Peer-reviewed journals and gray literature	• None
**Method**	• Reviews of quantitative studies (experimental, quasi-experimental, observational)	• Reviews of qualitative or theoretical studies
**Population**	• Parents or caregivers who take care of children at home	• Teachers or other adults in the community or who take care of children outside the home
**Interventions**	• Universal and selective interventions aimed at preventing VAC in the home	• Indicated interventions • Interventions targeting settings other than the home • Interventions that do not target VAC specifically
**Comparison (primary studies)**	• No intervention • Other interventions • Pre- and post-comparison	• No comparison group
**Outcomes**	• VAC in the home (reported by parents, children, others, administrative records, or direct assessment) • Modifiable drivers or direct risk factors for VAC	• VAC in settings other than the home (e.g., bullying) • Intimate partner violence • Community or other contextual violence
**Language**	• Any	• None
**Setting**	• Any	• None

We will exclude the studies meeting the following criteria: first, we will exclude reviews that focus exclusively on indicated interventions (i.e., those carried out in response to actual cases of VAC) instead of prevention interventions, and interventions that do not target VAC; second, interventions targeting VAC in settings other than the home, such as educational settings (e.g., bullying), community settings (e.g., crime or war), and others; third, reviews that do not include quantitative studies (e.g., qualitative studies); and fourth, reviews that focus on intimate partner violence (i.e., indirect VAC). We will exclude these types of violence considering that these are often not directed specifically towards the child (although we recognize them as a form of violence that can compromise children’s health and development and increase other forms of direct VAC [10, 24]). Finally, we will exclude studies published before 2000.

### Search strategy

We will search for reviews in PubMed, Embase (Elsevier), PsycInfo (EBSCO), and ERIC (EBSCO) using controlled vocabularies and keywords in titles and abstracts. For the searches, we will use specialized terms and keywords related to violence (e.g., abuse*, maltreatment, neglect), children (e.g., child, infant*, kid*), and intervention/prevention (e.g., program, strategy, interven*) (see Additional file 1 for search strategy). We will filter the results to search for studies published after 2000 and for reviews, following the search filter presented by Salvador-Oliván and coauthors [25]. We will also search for additional reviews that met the inclusion criteria in published systematic reviews of reviews [20, 22, 26].

### Screening and full text-review

We will export all reviews identified in the databases to the web-based software platform Covidence (https://www.covidence.org/) to ensure transparency and reproducibility of the overall decision process. As a first step, we will remove duplicates from the Covidence library. Subsequently, all titles and abstracts will be independently examined by two trained reviewers (JC and AS), considering the pre-established inclusion and exclusion criteria. All disagreements will be resolved by consensus through discussion among the reviewers. A third reviewer (DM) will offer input in case there are still unresolved conflicts. Articles meeting the inclusion criteria will continue to the full-text screening phase. We will retrieve and upload to Covidence all potentially relevant reviews, conducting similar procedures (i.e., independent review by JC and AS and input from DM for unresolved conflicts) to identify reviews that meet the inclusion criteria according to a review of the full text. Irrelevant articles will be flagged in Covidence, and reasons for exclusion will be itemized.

### Quality assessment

We will assess the methodological quality of the reviews using the A MeaSurement Tool to Assess systematic Reviews (AMSTAR) 2 critical appraisal tool of reviews that include randomized and non-randomized studies [27]. The AMSTAR 2 is a revised version of the AMSTAR instrument, which comprises 10 domains and 16 items regarding protocol registration before commencement of the review, adequacy of the literature search, justification for excluding studies, risk of bias on individual studies, and assessment of publication bias, among others. Two reviewers (JC and AS) will assess all included reviews using the AMSTAR 2.

### Data extraction

Two reviewers (JC and AS) will independently extract data for all included studies. The data will be extracted in Covidence using a pre-piloted data extraction template. We will extract information on the number of studies included, types of interventions considered (e.g., home visiting programs, sexual prevention strategies), main outcomes included in each review (e.g., specific forms of VAC or drivers of VAC), methodological criteria used, and main results (including main effects of the interventions and moderation or heterogeneous effects). Furthermore, we will extract basic characteristics like the geographic distribution of the primary studies included in the reviews, including country and region (East Asia and Pacific, Europe and Central Asia, Latin America and the Caribbean, Middle East and North Africa, North America, South Asia, Sub-Saharan Africa). Any disagreements in data extraction will be resolved through discussion between extractors, and DM will be consulted if any conflict is not resolved.

### Data synthesis

We will conduct a narrative review of the evidence, following the guidance presented by Popay and coauthors [28]. In particular, the narrative synthesis will comprise (1) a synthesis to organize the findings regarding the direction of effects of the interventions on specified outcomes, effect sizes, and evidence of moderation or heterogeneous effects; (2) an analysis of factors that might explain the differences in directions and size effects across studies; and (3) an assessment of the robustness and quality of the evidence. We will also conduct a quantitative assessment of the basic characteristics of the primary studies, including region and country. Using all the available information, we will critically assess the key findings from the literature, grouping studies by type of review (e.g., meta-analysis, systematic reviews) and identifying interventions that have been effective in preventing VAC or mitigating risk factors. We will synthesize the evidence regarding the prevention of violence and addressing the risk factors independently. Furthermore, we will assess the main limitations in the current literature as identified by the reviews, as well as discussing issues of external (i.e., generalizability) and internal (i.e., causality) validity to discuss the key next steps for research. Finally, we will draw policy implications from all available evidence to inform future efforts aimed at reducing VAC.

### Dissemination plans

We will submit the findings of the overview of reviews for publication to a peer-reviewed journal. We also expect to disseminate the findings from the overview of reviews in blogs and conferences or meetings related to the Lancet Commission on Gender-Based Violence and Maltreatment of Young People. Findings will also be included in the final report of the Lancet Commission on Gender-Based Violence and the Maltreatment of Young People and inform the broader recommendations of this Commission.

## Discussion

The SDGs established that VAC prevention is a global priority [18], considering that VAC constitutes a violation of children’s rights [29] and can disrupt children’s brain and skill development with long-lasting, costly consequences for individuals and societies [8, 9, 13, 30]. However, VAC is widespread around the globe, particularly in children’s homes, with millions of children experiencing physical punishment, psychological or emotional abuse, sexual abuse, and neglect, among others [3, 4]. Despite the prevalence and devastating consequences of VAC, few countries have implemented VAC prevention interventions and strategies with measurable outcomes, and there is an urgent need to systematize evidence on what works to prevent VAC at scale [19]. The systematic overview of reviews will aim to produce evidence on promising universal and selective interventions to prevent VAC in the home, as well as to discuss the amount and quality of existing evidence.

While the systematic overview of reviews will have several strengths, there will be some limitations that should be noted. First, we will conduct a narrative synthesis, not a quantitative summary of effect sizes, given the methodological heterogeneity of reviews to be included (e.g., systematic reviews, meta-analyses, rapid reviews). As such, we will not be able to assess the overall effect size for the impact of different interventions on VAC prevention or reduction in key risk factors (e.g., attitudes towards physical punishment). Another potential limitation is that included studies could vary in quality, with some studies having low methodological quality or poor reporting standards. However, we will employ the AMSTAR 2 tool and will critically assess the issues of external and internal validity and quality of reporting of included studies.

Moving forward, we will report any changes to the protocol while conducting the searches, screening and full-text review, and data extracting in PROSPERO and in the final manuscript to be submitted for publication.

Ultimately, the goals of this overview of reviews are to identify promising intervention approaches to prevent VAC in the home and to identify the strengths and limitations of the current body of evidence on this topic. In doing so, we will inform future research aimed at building effective strategies to prevent VAC globally. Most importantly, we will discuss the implications for policy and practice aimed at protecting children from violence so that individuals can achieve their developmental, health, and well-being potential.

## Supplementary Information

**Additional file 1.** PRISMA-P 2015 Checklist. Search code.

## Abbreviations

AMSTAR: A MeaSurement Tool to Assess systematic Reviews
PRISMA-P: Preferred Reporting Items for Systematic Reviews and Meta-Analysis for Protocols
PROSPERO: International Prospective Register of Systematic Reviews
SDGs: Sustainable Development Goals
UN: United Nations
VAC: Violence against children
