# Supplementary material for: Prevention of violence against children in the home: an overview of reviews protocol
Source: Syst Rev. 2022 Dec 15;11:272. doi: 10.1186/s13643-022-02153-6 (PMC9753069; doi:10.1186/s13643-022-02153-6)
Supplement: Supplementary file 1 — Additional file 1. PRISMA-P 2015 Checklist. Search code. [file 13643_2022_2153_MOESM1_ESM.docx]

**Prevention of violence against children in the home: An overview of reviews protocol**

**Supplemental files**

**Supplemental file 1: PRISMA-P 2015 Checklist**

| **Section/topic** | **#** | **Checklist item** | **Information reported** | | **Line number(s)** |
| --- | --- | --- | --- | --- | --- |
|  |  |  | **Yes** | **No** |  |
| **ADMINISTRATIVE INFORMATION** | | | | | |
| **Title** | | | | | |
| Identification | 1a | Identify the report as a protocol of a systematic review | X |  | 25 |
| Update | 1b | If the protocol is for an update of a previous systematic review, identify as such |  | X | N/A |
| **Registration** | 2 | If registered, provide the name of the registry (e.g., PROSPERO) and registration number in the Abstract | X |  | 40 |
| **Authors** | | | | | |
| Contact | 3a | Provide name, institutional affiliation, and e-mail address of all protocol authors; provide physical mailing address of corresponding author | X |  | 5-9 |
| Contributions | 3b | Describe contributions of protocol authors and identify the guarantor of the review | X |  | 249-251 |
| **Amendments** | 4 | If the protocol represents an amendment of a previously completed or published protocol, identify as such and list changes; otherwise, state plan for documenting important protocol amendments |  | X | N/A |
| **Support** | | | | | |
| Sources | 5a | Indicate sources of financial or other support for the review | X |  | 247-248 |
| Sponsor | 5b | Provide name for the review funder and/or sponsor | X |  | 247-248 |
| Role of sponsor/funder | 5c | Describe roles of funder(s), sponsor(s), and/or institution(s), if any, in developing the protocol | X |  | 248-250 |
| **INTRODUCTION** | | | | | |
| **Rationale** | 6 | Describe the rationale for the review in the context of what is already known | X |  | 44-95 |
| **Objectives** | 7 | Provide an explicit statement of the question(s) the review will address with reference to participants, interventions, comparators, and outcomes (PICO) | X |  | 96-104 |
| **METHODS** | | | | | |
| **Eligibility criteria** | 8 | Specify the study characteristics (e.g., PICO, study design, setting, time frame) and report characteristics (e.g., years considered, language, publication status) to be used as criteria for eligibility for the review | X |  | 115-146 |
| **Information sources** | 9 | Describe all intended information sources (e.g., electronic databases, contact with study authors, trial registers, or other grey literature sources) with planned dates of coverage | X |  | 148-155 |
| **Search strategy** | 10 | Present draft of search strategy to be used for at least one electronic database, including planned limits, such that it could be repeated | X |  | 148-155 |
| ***STUDY RECORDS*** | | | | | |
| Data management | 11a | Describe the mechanism(s) that will be used to manage records and data throughout the review | X |  | 157-159 |
| Selection process | 11b | State the process that will be used for selecting studies (e.g., two independent reviewers) through each phase of the review (i.e., screening, eligibility, and inclusion in meta-analysis) | X |  | 159-168 |
| Data collection process | 11c | Describe planned method of extracting data from reports (e.g., piloting forms, done independently, in duplicate), any processes for obtaining and confirming data from investigators | X |  | 178-188 |
| **Data items** | 12 | List and define all variables for which data will be sought (e.g., PICO items, funding sources), any pre-planned data assumptions and simplifications | X |  | 178-188 |
| **Outcomes and prioritization** | 13 | List and define all outcomes for which data will be sought, including prioritization of main and additional outcomes, with rationale | X |  | 128-132; 178-188 |
| **Risk of bias in individual studies** | 14 | Describe anticipated methods for assessing risk of bias of individual studies, including whether this will be done at the outcome or study level, or both; state how this information will be used in data synthesis | X |  | 169-176 |
| ***DATA*** | | | | | |
| **Synthesis** | 15a | Describe criteria under which study data will be quantitatively synthesized | X |  |  |
|  | 15b | If data are appropriate for quantitative synthesis, describe planned summary measures, methods of handling data, and methods of combining data from studies, including any planned exploration of consistency (e.g., *I* ^2^, Kendall’s tau) |  | X | N/A |
|  | 15c | Describe any proposed additional analyses (e.g., sensitivity or subgroup analyses, meta-regression) | X |  | N/A |
|  | 15d | If quantitative synthesis is not appropriate, describe the type of summary planned | X |  | 190-202 |
| **Meta-bias(es)** | 16 | Specify any planned assessment of meta-bias(es) (e.g., publication bias across studies, selective reporting within studies) | X |  | N/A |
| **Confidence in cumulative evidence** | 17 | Describe how the strength of the body of evidence will be assessed (e.g., GRADE) | X |  | 198-200 |

**Supplemental file 2: search code**

**PubMed**

((("2000/01/01"[Date - Publication] : "3000"[Date - Publication])) AND ((((((((("Violence"[Mesh] OR "Physical Abuse"[Mesh] OR "Child Abuse"[Mesh]) OR ("Gender-Based Violence"[Mesh] OR "Sexual Trauma"[Mesh] OR "Sex Offenses"[Mesh])) OR ("Emotional Abuse"[Mesh])) OR ("Exposure to Violence"[Mesh])) OR ("Armed Conflicts"[Mesh])) OR ("Social Deprivation"[Mesh])) OR (((physical[Title/Abstract] OR sexual[Title/Abstract] OR emotion*[Title/Abstract] OR psycholog*[Title/Abstract] OR medical[Title/Abstract] OR omission[Title/Abstract] OR commission[Title/Abstract] OR community [Title/Abstract]) AND (violen*[Title/Abstract] OR abuse*[Title/Abstract] OR neglect[Title/Abstract] OR harm[Title/Abstract] OR injur*[Title/Abstract] OR victim*[Title/Abstract])))) AND (("Adolescent"[Mesh] OR "Child"[Mesh] OR "Child, Preschool"[Mesh] OR "Infant"[Mesh] OR "Infant, Newborn"[Mesh] OR "Child, Orphaned"[Mesh] OR "Child, Unwanted"[Mesh] OR "Child, Abandoned"[Mesh]) OR (Child[Title/Abstract] OR Children[Title/Abstract] OR Kid[Title/Abstract] OR Kids[Title/Abstract] OR Youth[Title/Abstract] OR Teens[Title/Abstract] OR Teenager*[Title/Abstract] OR Adolescen*[Title/Abstract] OR young*[Title/Abstract] OR toddler*[Title/Abstract] OR infant*[Title/Abstract] OR baby[Title/Abstract] OR babies[Title/Abstract] OR orphan*[Title/Abstract] OR abandon*[Title/Abstract] OR unwanted[Title/Abstract]))) AND (("prevention and control" [Subheading] OR "Primary Prevention"[Mesh] OR "Psychosocial Intervention"[Mesh] OR "Policy"[Mesh]) OR (program*[Title/Abstract] OR framework*[Title/Abstract] OR strateg*[Title/Abstract] OR intervention*[Title/Abstract] OR prevent*[Title/Abstract] OR support*[Title/Abstract] OR promot*[Title/Abstract] OR lesson*[Title/Abstract] OR training*[Title/Abstract] OR legislat*[Title/Abstract] OR policy[Title/Abstract] OR policies[Title/Abstract])))) AND (((systematic* [ti] AND review [ti]) OR Systematic overview* [ti] OR Cochrane review* [ti] OR systemic review* [ti] OR scoping review [ti] OR scoping literature review [ti] OR mapping review [ti] OR Umbrella review* [ti] OR (review of reviews [ti] OR overview of reviews [ti]) OR meta-review [ti] OR (integrative review [ti] OR integrated review [ti] OR integrative overview [ti] OR meta-synthesis [ti] OR metasynthesis [ti] OR quantitative review [ti] OR quantitative synthesis [ti] OR research synthesis [ti] OR meta-ethnography [ti]) OR Systematic literature search [ti] OR Systematic literature research [ti] OR meta-analyses [ti] OR metaanalyses [ti] OR metaanalysis [ti] OR meta-analysis [ti] OR meta-analytic review [ti] OR meta-analytical review [ti] OR meta-analysis [pt] OR ((search* [tiab] OR medline [tiab] OR pubmed [tiab] OR embase [tiab] OR Cochrane [tiab] OR scopus [tiab] or web of science [tiab] OR sources of information [tiab] OR data sources [tiab] OR following databases [tiab]) AND (study selection [tiab] OR selection criteria [tiab] OR eligibility criteria [tiab] OR inclusion criteria [tiab] OR exclusion criteria [tiab])) OR systematic review [pt]) NOT (letter [pt] OR editorial [pt] OR comment [pt] OR case reports [pt] OR historical article [pt] OR report [ti] OR protocol [ti] OR protocols [ti] OR withdrawn [ti] OR retraction of publication [pt] OR retraction of publication as topic [mesh] OR retracted publication [pt] OR reply [ti] OR published erratum [pt]))

**Embase (Elsevier)**

#14 #12 AND #13

#13 systematic*:ti AND review:ti OR 'systematic overview*':ti OR 'cochrane review*':ti OR 'systemic review*':ti OR 'scoping review':ti OR 'scoping literature review':ti OR 'mapping review':ti OR 'umbrella review*':ti OR 'review of reviews':ti OR 'overview of reviews':ti OR 'meta review':ti OR 'integrative review':ti OR 'integrated review':ti OR 'integrative overview':ti OR 'meta synthesis':ti OR metasynthesis:ti OR 'quantitative review':ti OR 'quantitative synthesis':ti OR 'research synthesis':ti OR 'meta ethnography':ti OR 'systematic literature search':ti OR 'systematic literature research':ti OR 'meta analyses':ti OR metaanalyses:ti OR metaanalysis:ti OR 'meta analysis':ti OR 'meta-anaytic review':ti OR 'meta-analytical review':ti OR ((search:ab,ti OR medline:ab,ti OR pubmed:ab,ti OR embase:ab,ti OR cochrane:ab,ti OR scopus:ab,ti OR 'web of science':ab,ti OR 'sources of information':ab,ti OR 'data sources':ab,ti OR 'following databases':ab,ti) AND ('study selection':ab,ti OR 'selection criteria':ab,ti OR 'eligibility criteria':ab,ti OR 'inclusion criteria':ab,ti OR 'exclusion criteria':ab,ti)) OR 'systematic review'/de

#12 #3 AND #6 AND #9 AND [embase]/lim AND [2000-2022]/py

#11 #3 AND #6 AND #9 AND [embase]/lim

#10 #3 AND #6 AND #9

#9 #7 OR #8

#8 program*:ti,ab,kw OR framework*:ti,ab,kw OR strateg*:ti,ab,kw OR intervention*:ti,ab,kw OR prevent*:ti,ab,kw OR support*:ti,ab,kw OR promot*:ti,ab,kw OR lesson*:ti,ab,kw OR training*:ti,ab,kw OR legislat*:ti,ab,kw OR policy:ti,ab,kw OR policies:ti,ab,kw

#7 'prevention and control'/exp OR 'primary prevention'/exp OR 'psychosocial intervention'/exp OR 'policy'/exp

#6 #4 OR #5

#5 child:ti,ab,kw OR children:ti,ab,kw OR kid:ti,ab,kw OR kids:ti,ab,kw OR youth:ti,ab,kw OR teens:ti,ab,kw OR teenager*:ti,ab,kw OR adolescen*:ti,ab,kw OR young*:ti,ab,kw OR toddler*:ti,ab,kw OR infant*:ti,ab,kw OR baby:ti,ab,kw OR babies:ti,ab,kw OR orphan*:ti,ab,kw OR abandon*:ti,ab,kw OR unwanted:ti,ab,kw

#4 'adolescent'/exp OR 'child'/exp OR 'infant'/exp OR 'newborn'/exp OR 'preschool child'/exp OR 'orphaned child'/exp OR 'abandoned child'/exp OR 'unwanted child'/exp

#3 #1 OR #2

# 2 ((physical OR sexual OR emotion* OR psycholog* OR medical OR mission OR commission OR community) NEAR/2 (violen* OR abus* OR neglect OR harm OR injur* OR victim*)):ti,ab,kw

# 1 'violence'/exp OR 'physical abuse'/exp OR 'child abuse'/exp OR 'gender based violence'/exp OR 'sexual trauma'/exp OR 'sexual crime'/exp OR 'emotional abuse'/exp OR 'exposure to violence'/exp OR 'war'/exp OR 'neglect'/exp OR 'social isolation'/exp

**ERIC (EBSCO)**

S15 S11 AND S14

S14 S12 OR S13

S13 TI (((search OR medline OR pubmed OR embase OR Cochrane OR scopus OR "web of science" OR "sources of information" OR "data sources" OR "following databases") AND ("study selection" OR "selection criteria" OR "eligibility criteria" OR "inclusion criteria" OR "exclusion criteria")) ) OR AB ( ((search OR medline OR pubmed OR embase OR Cochrane OR scopus OR "web of science" OR "sources of information" OR "data sources" OR "following databases") AND ("study selection" OR "selection criteria" OR "eligibility criteria" OR "inclusion criteria" OR "exclusion criteria")))

S12 TI (systematic* AND review) OR "systematic overview*" OR "cochrane review*" OR "systemic review*" OR "scoping review" OR "scoping literature review" OR "mapping review" OR "umbrella review*" OR "review of reviews" OR "overview of reviews" OR meta-review OR "integrative review" OR "integrated review" OR "integrative overview" OR meta-synthesis OR metasynthesis OR "quantitative review" OR "quantitative synthesis" OR "research synthesis" OR meta-ethnography OR "systematic literature search" OR "systematic literature research" OR meta-analyses OR metaanalyses OR metaanalysis OR meta-analysis OR "meta-analytic review" OR "meta-analytical review"

S11 S10 AND (DT 2000-2022)

S10 S3 AND S6 AND S9

S9 S7 OR S8

S8 TI ( program* OR framework* OR strateg* OR intervention* OR prevent* OR support* OR promot* OR lesson* OR training* OR legislat* OR policy OR policies ) OR AB ( program* OR framework* OR strateg* OR intervention* OR prevent* OR support* OR promot* OR lesson* OR training* OR legislat* OR policy OR policies ) OR KW ( program* OR framework* OR strateg* OR intervention* OR prevent* OR support* OR promot* OR lesson* OR training* OR legislat* OR policy OR policies )

S7 ((DE "Prevention" OR DE "Crime Prevention") OR (DE "Intervention" OR DE "Crisis Intervention")) OR (DE "Policy")

S6 S4 OR S5

S5 TI ( Child OR Children OR Kid OR Kids OR Youth OR Teens OR Teenager* OR Adolescen* OR young* OR toddler* OR infant* OR baby OR babies OR orphan* OR abandon* OR unwanted ) OR AB ( Child OR Children OR Kid OR Kids OR Youth OR Teens OR Teenager* OR Adolescen* OR young* OR toddler* OR infant* OR baby OR babies OR orphan* OR abandon* OR unwanted ) OR KW ( Child OR Children OR Kid OR Kids OR Youth OR Teens OR Teenager* OR Adolescen* OR young* OR toddler* OR infant* OR baby OR babies OR orphan* OR abandon* OR unwanted)

S4 (DE "Adolescents" OR DE "Children") OR (DE "Infants" OR DE "Neonates")

S3 S1 OR S2

S2 TI (((Physical OR sexual OR emotion* OR psycholog* OR medical OR mission OR commission OR community) N2 (violen* OR abus* OR neglect OR harm OR injur* OR victim*)) ) OR AB ( ((Physical OR sexual OR emotion* OR psycholog* OR medical OR mission OR commission OR community) N2 (violen* OR abus* OR neglect OR harm OR injur* OR victim*)) ) OR KW ( ((Physical OR sexual OR emotion* OR psycholog* OR medical OR mission OR commission OR community) N2 (violen* OR abus* OR neglect OR harm OR injur* OR victim*)))

S1 ( DE "Violence" OR DE "Family Violence" OR DE "Child Abuse" ) OR DE "Sexual Abuse" OR DE "Antisocial Behavior" OR DE "War" OR DE "Child Neglect"

**APA PsycInfo (EBSCO)**

S16 S12 AND S15

S15 S13 OR S14

S14 TI (((search OR medline OR pubmed OR embase OR Cochrane OR scopus OR "web of science" OR "sources of information" OR "data sources" OR "following databases") AND ("study selection" OR "selection criteria" OR "eligibility criteria" OR "inclusion criteria" OR "exclusion criteria")) ) OR AB ( ((search OR medline OR pubmed OR embase OR Cochrane OR scopus OR "web of science" OR "sources of information" OR "data sources" OR "following databases") AND ("study selection" OR "selection criteria" OR "eligibility criteria" OR "inclusion criteria" OR "exclusion criteria")))

S13 TI (systematic* AND review) OR "systematic overview*" OR "cochrane review*" OR "systemic review*" OR "scoping review" OR "scoping literature review" OR "mapping review" OR "umbrella review*" OR "review of reviews" OR "overview of reviews" OR meta-review OR "integrative review" OR "integrated review" OR "integrative overview" OR meta-synthesis OR metasynthesis OR "quantitative review" OR "quantitative synthesis" OR "research synthesis" OR meta-ethnography OR "systematic literature search" OR "systematic literature research" OR meta-analyses OR metaanalyses OR metaanalysis OR meta-analysis OR "meta-analytic review" OR "meta-analytical review"

S12 S11 AND DT 2000-2022

S11 S3 AND S7 AND S10

S10 S8 OR S9

S9 TI ( program* OR framework* OR strateg* OR intervention* OR prevent* OR support* OR promot* OR lesson* OR training* OR legislat* OR policy OR policies ) OR AB ( program* OR framework* OR strateg* OR intervention* OR prevent* OR support* OR promot* OR lesson* OR training* OR legislat* OR policy OR policies )

S8 (DE "Violence Prevention") OR (DE "Intervention")

S7 S4 OR S5 OR S6

S6 TI ( Child OR Children OR Kid OR Kids OR Youth OR Teens OR Teenager* OR Adolescen* OR young* OR toddler* OR infant* OR baby OR babies OR orphan* OR abandon* OR unwanted ) OR AB ( Child OR Children OR Kid OR Kids OR Youth OR Teens OR Teenager* OR Adolescen* OR young* OR toddler* OR infant* OR baby OR babies OR orphan* OR abandon* OR unwanted )

S5 AG ("adolescence" OR "infancy" OR "neonatal" OR "preschool age" OR "school age" OR "childhood" )

S4 DE "Orphans"

S3 S1 OR S2

S2 TI ( ((Physical OR sexual OR emotion* OR psycholog* OR medical OR mission OR commission OR community) N2 (violen* OR abus* OR neglect OR harm OR injur* OR victim*)) ) OR AB ( ((Physical OR sexual OR emotion* OR psycholog* OR medical OR mission OR commission OR community) N2 (violen* OR abus* OR neglect OR harm OR injur* OR victim*)))

S1 (((DE "Child Abuse") OR (DE "Physical Abuse")) OR (DE "Violence") ) OR ( (DE "Sexual Violence") OR (DE "Sex Offenses" OR DE "Incest" OR DE "Sexual Abuse") ) OR ( (DE "Emotional Abuse")) OR DE "Exposure to Violence" OR DE "War" OR ( DE "Child Neglect" OR (DE "Social Deprivation"))
